# Molecular mechanism of circadian rhythmicity of seizures in temporal lobe epilepsy

**DOI:** 10.3389/fncel.2012.00055

**Published:** 2012-11-23

**Authors:** Chang-Hoon Cho

**Affiliations:** Epilepsy Research Laboratory, Department of Pediatrics, Children's Hospital of PhiladelphiaPhiladelphia, PA, USA

**Keywords:** epilepsy, circadian, rhythmicity, mTOR, CLOCK, hippocampus, SCN, subiculum

## Abstract

The circadian pattern of seizures in people with epilepsy (PWE) was first described two millennia ago. However, these phenomena have not received enough scientific attention, possibly due to the lack of promising hypotheses to address the interaction between seizure generation and a physiological clock. To propose testable hypotheses at the molecular level, interactions between circadian rhythm, especially transcription factors governing clock genes expression, and the mTOR (mammalian target of rapamycin) signaling pathway, the major signaling pathway in epilepsy, will be reviewed. Then, two closely related hypotheses will be proposed: (1) Rhythmic activity of hyperactivated mTOR signaling molecules results in rhythmic increases in neuronal excitability. These rhythmic increases in excitability periodically exceed the seizure threshold, displaying the behavioral seizures. (2) Oscillation of neuronal excitability in SCN modulates the rhythmic excitability in the hippocampus through subiculum via long-range projections. Findings from published results, their implications, and proposals for new experiments will be discussed. These attempts may ignite further discussion on what we still need to learn about the rhythmicity of spontaneous seizures.

## Introduction

Circadian rhythmicity of epileptic seizures was described over 2000 years ago, and modern scientific studies were conducted in the late nineteenth century (Gowers, 1885; Wilson and Reynolds, 1990). In these early studies, approximately two thirds of people with epilepsy (PWE) showed circadian patterns of epileptic episodes (diurnal, nocturnal, and the rest categorized as “diffuse” type—seizures occur randomly without a certain pattern). Diurnal seizures were known to cluster in wakefulness or in the late afternoon, while nocturnal seizures occurred frequently at bedtime and in the early morning before awakening (Langdon-Down and Brain, 1929; Gfiffiths and Fox, 1938). This circadian pattern of seizures in PWE tends to be well preserved. In sleep-related studies, non-rapid eye movement is typically associated with the increase in epileptiform discharges and seizures in nocturnal cases (Shouse et al., 2000). Circadian rhythm has a tremendous influence on sleep (and vice versa) and the relationship between epilepsy and the sleep-wake cycle has been actively studied (Bazil and Walczak, 1997; Matos et al., 2011; Zarowski et al., 2011). Therefore, this article will focus mainly on the relationship between intrinsic circadian rhythm and epilepsy.

## Circadian rhythms

Circadian rhythms are endogenously controlled 24 h (approximately) cycles of behavioral and physiological processes such as sleep-wake cycle, hormonal production (e.g., cortisol, glucocorticoid, and melatonin), and regulation of body temperature and blood pressure (Hastings et al., 2007; Albrecht, 2012). The circadian clock of most organisms is controlled by both photic (light–dark cycle of the environment) and non-photic (such as daily feeding or behavioral activities) stimuli (Reebs and Mrosovsky, 1989; Rosenwasser and Dwyer, 2001; van Oosterhout et al., 2012).

## Molecular mechanism of circadian gene regulation

**Figure 1:**
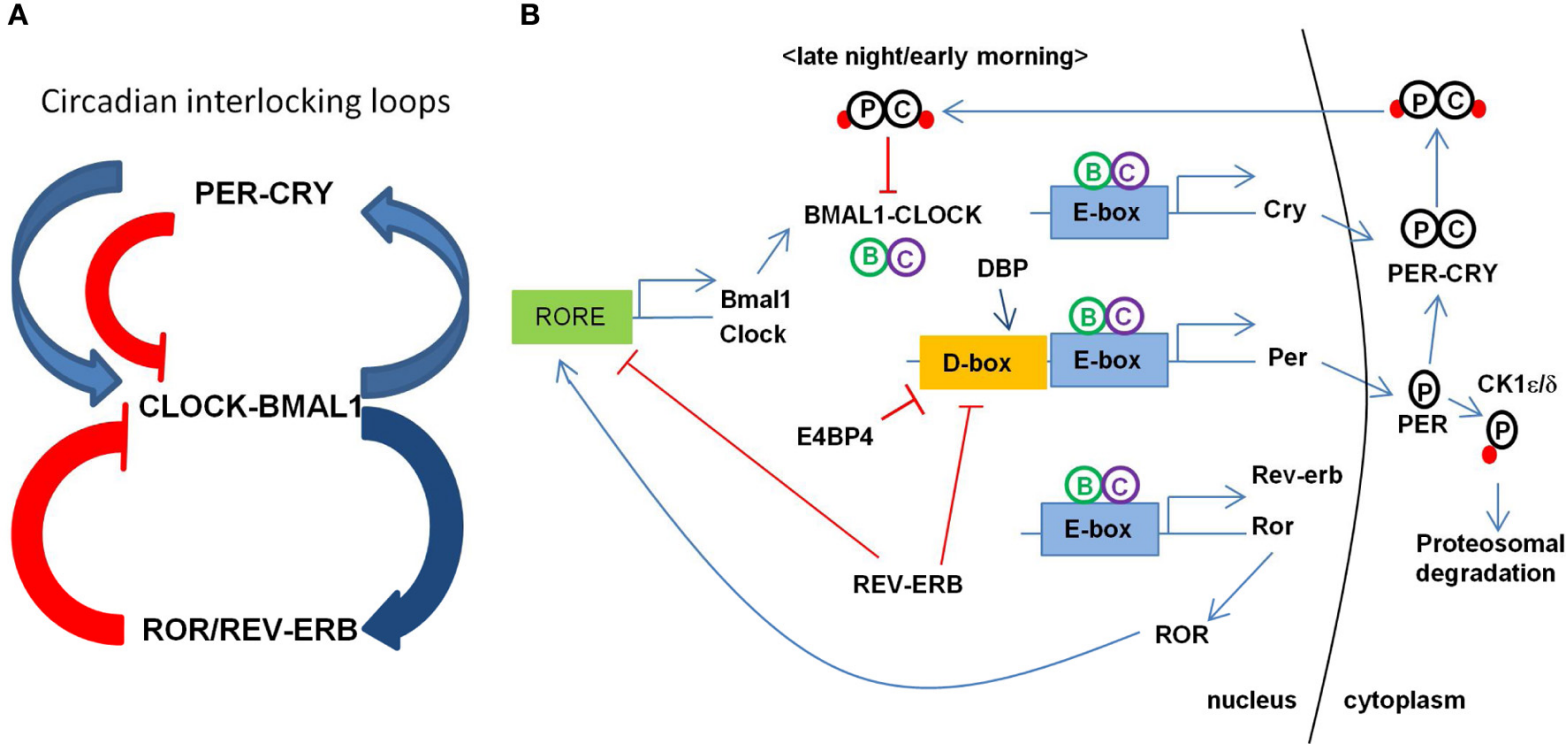
**Transcriptional regulation circuit of clock genes in mammals. (A)** Circadian interlocking loops show that a primary loop of CLOCK-BMAL1 and PER-CRY complexes and an additional feedback loop of ROR/REV-ERB, conferring a tight transcriptional regulation. The blue arrows indicate the transcriptional activation and the red lines indicate the inhibiting activity of the targets. ROR as an activator and REV-ERB as a repressor regulate the expression of BMAL1. **(B)** Transcription of BMAL1 and CLOCK is regulated by ROR and REV-ERB through binding RORE elements at their promoters. CLOCK and BMAL1 activates the expression of CRY, PER, REV-ERB, ROR, and other CCGs (clock controlled genes) through binding to E-box element at their promoters. CRY-PER complex is phosphorylated and transported back to the nucleus inhibiting the CLOCK-BMAL1 activity. PER is phosphorylated to degrade through proteosomal pathway via CK1ε/δ.

Maintenance of the circadian clock involves coordinated feedback regulation of transcription and translation of CLOCK genes to achieve the oscillatory levels of activators and repressors (Figure 1; for the review, see Albrecht, 2012; Zheng and Sehgal, 2012). In a primary loop, CLOCK and BMAL1 (also known as ARNTL) form a large complex in the cytoplasm and translocate to the nucleus after being phosphorylated by protein kinases (e.g., CK1ε/δ) to activate the transcription of PERIOD (PER1, PER2, and PER3) and CRYPTOCHROME (CRY1 and CRY2) genes. The PER-CRY complex then subsequently binds to CLOCK-BMAL1 complex to repress their transcriptions. PER and CRY are degraded through the ubiquitin-proteosomal pathway (e.g., FBXL3-dependent) and this whole process takes about a 24 h cycle. An additional feedback loop is at work with nuclear hormone receptors such as ROR (RORa, RORb, and RORc) and REV-ERBα/β to modulate the expression of clock-controlled genes (CCGs) and clock-modulated genes. Several circadian modulators such as DEC1/2 (also called BHLHE40/41), DBP, and E4BP4 (also called NFIL3) provide the additional level of circadian regulation. In the promoter region of core CLOCK genes and CCGs, E-box elements are recognized by BMAL1-CLOCK, D-box elements by DBP-E4BP4, and REV-ERBα/ROR-regulatory elements (RORE) by ROR. CLOCK, BMAL1, and PER1 are acetylated in response to the environmental stimuli to adjust the activity of core clock proteins. Changes or disruption in this multi-step regulation influences the 24-h period by shortening or lengthening it.

## Circadian regulation of ion channels and membrane excitability

Neurotransmitter receptors and ion channels have been shown to have rhythmic expression and activity under circadian regulation (Kafka et al., 1986; Ko et al., 2009). Radioactive ligand binding assay of several neurotransmitter receptors in rat brains showed that the cortex has the highest variation and that the cerebellum has the lowest. Hippocampus has circadian patterns of ligand binding activities of α1 adrenergic and benzodiazepine receptors (Kafka et al., 1986). Although the studies have been limited mostly to the visual system (photoreceptors, retinal neurons, and suprachiasmatic nucleus), cGMP-gated ion channel, T- and L-type Ca channels, and voltage-gated K channels have been shown to be under circadian control (Ko et al., 2009). Clock gene products are involved in rhythmicity of membrane excitability and electrical activities mostly due to changes in potassium conductance (Belle et al., 2009; Itri et al., 2010). The expression of pyridoxal kinase, an enzyme involved in metabolism of pyridoxal phosphate and neurotransmitters (e.g., serotonin and dopamine), has shown to be regulated by circadian PAR bZIP transcription factors (Gachon et al., 2004). Thus, circadian rhythm modulates neuronal excitability at the multiple levels, may trigger the hyperexcitability out of delicate control.

## Epilepsy

Neuronal excitability is homeostatically controlled between excitatory and inhibitory drives in the nervous system. Hyperexcitability, caused by the disruption of this delicate balance at the microcircuit level, may trigger the excessively synchronized electrical discharges of neurons in the brain which can manifest as epileptic seizures (Bertram, 2008). As a global health issue, epilepsy affects ~1% of the general population (World Health Organization, 2005). Temporal lobe epilepsy (TLE), especially, is often pharmacologically refractory and is the most common type of acquired epilepsy that involves the hippocampus, entorhinal cortex, and amygdala (Bertram, 2008).

## Circadian pattern of epileptic seizures in human and animal models

The circadian pattern of seizures tends to be well preserved over the years in individuals and some PWE experience the episodes at the certain time of the day. However, the majority of one type over the other (nocturnal vs. diurnal) in the epileptic population is not always consistent in the literature (Méndez and Radtke, 2001). This may be the result of heterogeneity between the cohorts recruited for each study. For instance, Gowers and Patry described independently that seizures are more frequent during the daytime than the night among the PWE they have observed (Gowers, 1885; Patry, 1931). On the contrary, Janz and Hopkins independently found that nocturnal seizures are more prevalent than the diurnal ones (Hopkins, 1933; Janz, 1962). It is not straightforward to compare their findings because PWE were not classified based on seizure type, age, or other possibly important factors (e.g., comorbidity) of PWE in individual study. Additional studies, possibly collaborations at multiple epilepsy clinics, with a standardized protocol to recruit PWE, a clear classification of epilepsy/seizure types, and continuous monitoring and data analysis, are needed in order to provide a better picture of the phenomena.

In studies with a small cohort, epileptic seizures with circadian rhythmicity seem to be dependent on the origin and type of seizures (Hofstra et al., 2009a,b; Zarowski et al., 2011). For example, de Weerd and colleagues used the video-EEG monitoring to describe that complex partial and temporal seizures in adults have the peak activity during 11:00–17:00 h period, and parietal seizures occurs more frequently during 17:00–23:00 period. In addition, frontal seizures showed the age-specific peak activities during 23:00–5:00 period in adults and 17:00–23:00 in children (Hofstra et al., 2009a,b). Children with generalized seizures showed that tonic and tonic-clonic seizures were more frequently observed during sleep, whereas clonic, absence, atonic, and myoclonic types of seizures have various peak times in wakefulness (Zarowski et al., 2011).

Animal models of epilepsy also display circadian patterns of seizures (Fenoglio-Simeone et al., 2009; Tchekalarova et al., 2010; Matzen et al., 2012). Chronically epileptic KCNA1 null mice have peak seizure occurrence early in the morning (at Zeitgeber 2.3), and seizure occurrence and rest-activity rhythm are inversely correlated. KCNA1 null mice have a longer circadian period than wild-type mice, and they are either phase-delayed or -advanced (Fenoglio-Simeone et al., 2009). A kainate rat model of TLE showed the higher seizure prevalence during the day and those placed in constant darkness (light-deprived) displayed spontaneous seizures that still followed a circadian pattern, suggesting that there is an endogenously mediated circadian pattern (Quigg, 2000; Tchekalarova et al., 2010). This diurnal tendency has been also found in several different epilepsy models (Quigg et al., 1998; Arida et al., 1999; Hellier and Dudek, 1999; Nissinen et al., 2000; Stewart and Leung, 2003; Raedt et al., 2009). Human and rodent models of TLE showed higher seizure prevalence during the day regardless of the species difference in the sleep-wake cycle. No direct association has been established between abnormalities (e.g., mutation) of major CLOCK gene products and epilepsy.

## Chronotherapy for epilepsy

Circadian influence on the dynamics and kinetics of medications in individuals is important in drug efficacy, and it needs to be monitored for improved treatment (Ohdo et al., 2010; Paschos et al., 2010). Differential dosing of medication for patients with cancer, asthma, hypertension, or diabetes based on individuals' circadian patterns have been shown effective (Lévi et al., 2010; Gimble et al., 2011; Hermida et al., 2011). Differential dosing of anticonvulsants to relieve the seizure has been reported to be more effective when the timing of drug intake is adjusted to the day-night shift (Yegnanarayan et al., 2006; Guilhoto et al., 2011).

## The mTOR pathway in epilepsy and circadian regulation

The mTOR signaling pathway play major roles in regulating gene transcription and protein translation and it has been deeply involved in several physiological and pathological conditions (Laplante and Sabatini, 2012). This pathway has also been recognized as a major signaling pathway in acquired epilepsies as well as a few mutation-based epilepsies (see Cho, 2011 for the detail). Rapamycin, an mTORC1 kinase inhibitor, blocks epileptogenesis and reduces the seizure frequency in the pilocarpine/kainate-injected rats when repeatedly administrated (Buckmaster et al., 2009; Zeng et al., 2009; Huang et al., 2010). Rapamycin also suppresses axonal sprouting of somatostatin-positive interneurons in the dentate/hilus (Buckmaster and Wen, 2011). A study shows that the sclerotic hippocampi of human specimen with refractory TLE, as well as kainate mouse model, have over-activated mTOR markers in reactive astrocytes (Sha et al., 2012; Sosunov et al., 2012).

Relatively high levels of basal mTOR activity have been reported in SCN. Its maximal activity occurs during the subjective day and minimal activity during the late subjective night (Cao and Obrietan, 2010; Cao et al., 2010). Phosphorylated (activated) S6, a ribosomal protein important in protein synthesis and a downstream target of mTORC1, oscillates synchronously with PER1 expression, and photic stimulation elicits a coordinate upregulation of PER1 and mTOR activation in SCN (Cao et al., 2010). Interestingly, some of the key molecules in the mTOR pathway have been shown to be regulated in circadian manner (Zhang et al., 2009). By genome-wide RNAi screening in a model cell line, 17 gene products have been identified as strong circadian clock modifiers in period length and amplitude. These proteins showed a “network effect”—leading to dynamic changes in protein-protein interaction, phosphorylation, trans-activation, or trans-repression when affected. An insulin signaling pathway (mTOR-dependent) has been shown to regulate the circadian clock (Zhang et al., 2009).

**Figure 2:**
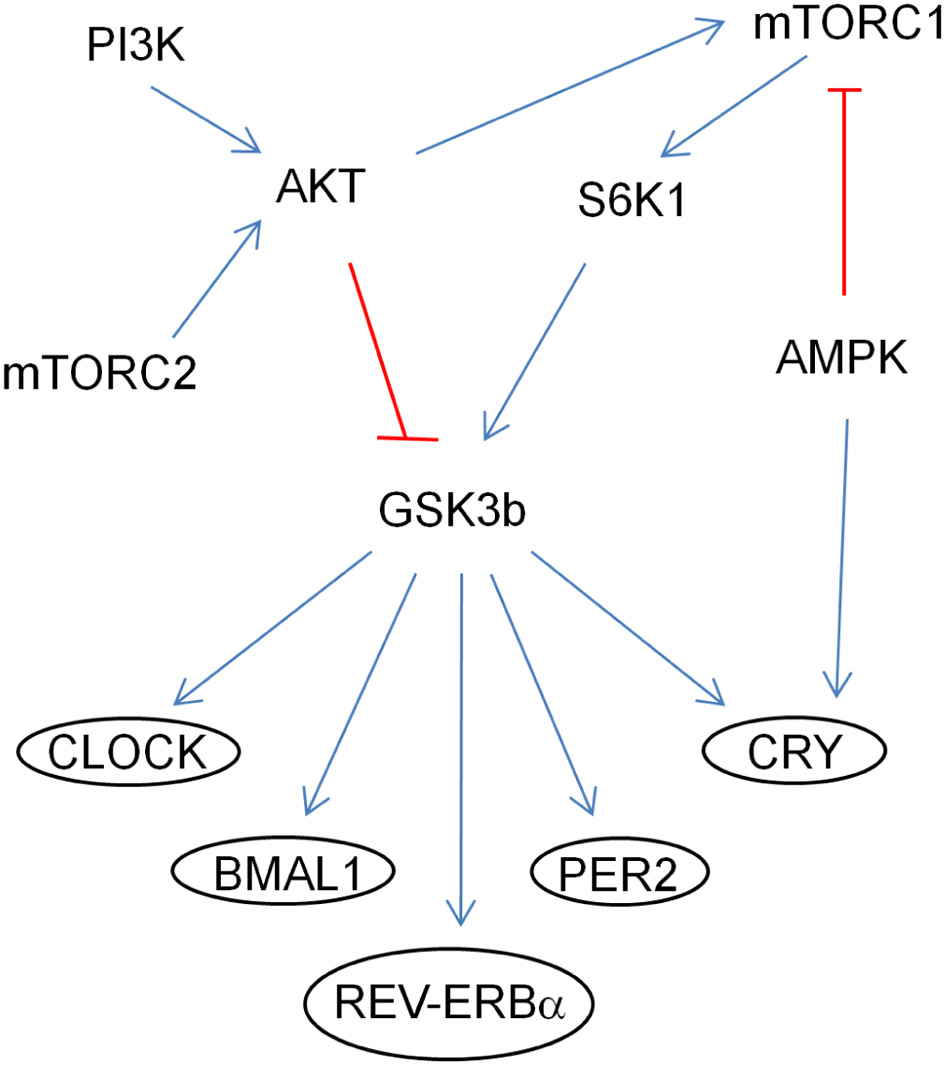
**Regulation of CLOCK proteins by the mTOR pathway through GSK3β.** The arrows (in blue) indicate activation of the targets and the ones (in red) indicate inhibition by phosphorylation.

In addition, by genetically manipulating signaling molecules in *Drosophila in vivo*, PTEN-AKT-Rheb-TOR-S6K pathway has been shown to affect the circadian period (Zheng and Sehgal, 2010). SGG (*Drosophila* homolog of GSK3β) is phosphorylated by AKT and S6K1 and it phosphorylates TIMELESS (*Drosophila* homolog of CRYPTOCHROME), modulating its nuclear translocation with PERIOD (Figure 2; Martinek et al., 2001; Papadopoulou et al., 2004; Zhang et al., 2006). GSK3β may also modulate CLOCK, *BMAL1*, and REV-ERBα (Yin et al., 2006; Spengler et al., 2009; Sahar et al., 2010). Conditional knockout PTEN mice driven by the NSE-Cre promoter have a lengthened period (Ogawa et al., 2007). PI3K and mTOR are periodic and cyclic, and IRS and 4EBP1 are cyclic (Zhang et al., 2009). High-fat diet lengthened the locomotor activity rhythm and modulated CLOCK genes at the molecular level in mice (Kohsaka et al., 2007).

The catalytic subunits (α1 and α2) of AMP protein kinase (AMPK), which is an upstream regulator of mTOR kinase, regulate circadian rhythms (Um et al., 2011). AMPK phosphorylates and modulates the activity of CRYPTOCHROME (Lamia et al., 2009). Ketogenic diet (KD), a strict dietary plan to reduce the frequency and severity of seizure episodes in some population of epileptic patients, has been shown to be mTOR-dependent (McDaniel et al., 2011). In epileptic KCNA1 null mice, KD reduces frequency and periodicity of seizures, and it also improves diurnal rhythmicity (Fenoglio-Simeone et al., 2009). Since KD works through mTOR pathway, it will be interesting to see if mTOR inhibitors will have a beneficial effect on this mouse model. Therefore, it is a plausible that the circadian rhythmicity of seizure episode is mediated by the fluctuation in activity of the mTOR signaling molecules. However, there is no direct evidence so far to support this hypothesis. Examining the circadian pattern of activity and expression of mTOR signaling molecules in epilepsy models, and studying the behavioral rhythm of null mice of those molecules will be valuable.

## Transcription factors governing the circadian clock as molecular links to epilepsy

There are over 2000 CCGs reported in mammals from the public microarray studies, and more than 20 transcription factors are found or suggested to be important in circadian expression patterns of CCGs via a large scale promoter analysis (Bozek et al., 2009). There are several findings to suggest that these transcription factors have been involved in epilepsy.

A GC-rich motif, EGR is significantly overrepresented in the promoter region of CCGs (Bozek et al., 2009). Increased levels of EGR-1 and EGR-2, which bind to the EGR element, have been reported in the neocortex of epileptic patients (Rakhade et al., 2005). AP1, a promising circadian regulator, has the high level in cerebral cortex and hippocampus of epileptic E1 mice (Yoneda et al., 1993). STAT3, which regulates the expression of GABAα1 receptor subunit, has been shown to be activated (phosphorylated) in GFAP-positive astrocytes in the hippocampus in pilocarpine-induced model (Lund et al., 2008; Xu et al., 2011). SP1 has a long-lasting increased activity in kainate-induced epilepsy model, and neonatal epilepsy-associated KCNQ2 and KCNQ3 genes are activated by SP1 (Feng et al., 1999; Mucha et al., 2010). DBP has the increased level in cerebrospinal fluid has been found in TLE patients and its overepxression in mice increased the seizure susceptibility (Klugmann et al., 2006; Xiao et al., 2009). In contrast, triple knockout mice of circadian PAR bZIP transcription factors (DBP, HLF, and TEF) exhibit epileptic symptoms (Gachon et al., 2004).

XBP1 (X-box-binding proteins 1), a basic leucine zipper family transcription factor, is recently identified as one of light-inducible genes in chicken pineal gland, and its spliced form has circadian pattern of gene expression (Hatori et al., 2011). The splicing and expression of XBP1 is increased when the mTOR pathway is activated, affecting XBP1-targeted genes (Pfaffenbach et al., 2010). Its increased expression and activation has been shown in hippocampi of epileptic patients (Liu et al., 2011). SREBP1 (Sterol regulatory element binding protein) is a transcription factor controlling expression of genes involved in lipid and cholesterol biosynthesis (Laplante and Sabatini, 2009; Porstmann et al., 2009). The mTORC1 phosphorylates SREBP1 to upregulate the expression of its target genes (Porstmann et al., 2008). Its expression follows the circadian pattern as it is XBP1 and mTOR-dependent (Hatori et al., 2011). One of SREBP1 downstream targets, stearoyl-CoA desaturase 1 has been shown to be upregulated in human cortical specimen of TLE (Arion et al., 2006).

In addition to the proteins that were mentioned above, Oligophrenin-1, PAM, and the GABA_A_ receptor β2 subunit are linked to epilepsy and circadian behavior (Tentler et al., 1999; Bergmann et al., 2003; Arion et al., 2006; Yin et al., 2010). Specifically, Oligophrenin-1 interacts with Rev-erbα, a nuclear receptor involved in regulation of the circadian clock, and regulates the oscillatory expression of a clock gene BMAL1 in the hippocampus (Valnegri et al., 2011). Therefore, abnormal activity of these transcription factors controlling circadian rhythm is also deeply involved in epilepsy. Several questions remain. Is the increased expression of these transcription factors sufficient to lower the seizure threshold and/or make the neurons hyperexcitable? Will reducing these factors in the epileptic animals (e.g., using siRNA technique) decrease the seizure frequency or even change the circadian pattern of seizures? Are the expression and/or activation of these factors mTOR pathway dependent? Will the altered activity of these factors be reversed when the rapamycin or anticonvulsants are administered?

## Functional connection between hippocampus and SCN

**Figure 3:**
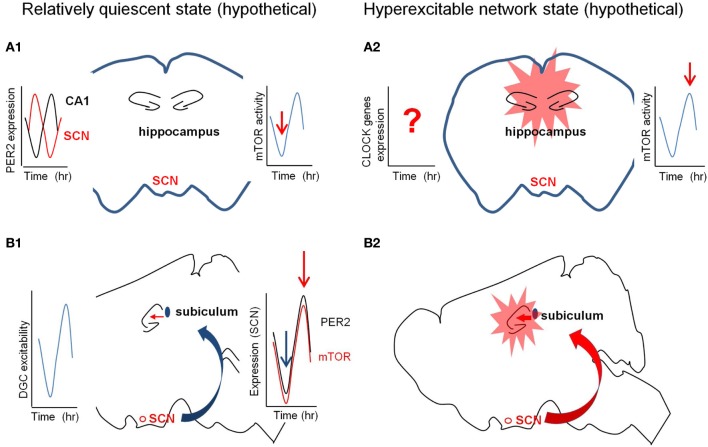
**Hypothetical diagrams of two different network excitability states.** The left panel (in **A1** and **B1**) is in quiescent state and the right (in **A2** and **B2**) is in hyperexcitable state. **(A)** The circadian fluctuation of the expression and/or activity of mTOR signaling molecules in the hippocampus may determine the quiescent or hyperexcitable states of the hippocampus prone to epileptic seizures. The red arrows in the right graphs from the cartoon of the coronal section indicate the hypothetical activation states of mTOR pathway (**A1** and **A2**). The left graph in **(A1)** indicates the out-of-phase rhythmic expression of PER2 in the SCN and CA1. **(B)** The circadian fluctuation of the SCN inputs to hippocampus via subiculum may regulate the excitability states of the hippocampus. The left graph in **(B1)** shows the rhythmic excitability of the dentate gyrus and the right graph in B2 signifies the synchronized rhythmicity of PER2 and mTOR signaling molecules. SCN, suprachiasmatic nucleus.

In the hippocampus, the activity (and/or the expression level) of several memory-related proteins has been shown to oscillate in the circadian manner (e.g., adenylyl cyclases, ERK/MAPK, Ras, MEK, and CREB) (Eckel-Mahan et al., 2008). LTP, field EPSP slope, and population spike in the dentate are greater during the dark phase than the light phase when medial perforant path was stimulated (Harris and Teyler, 1983; Bowden et al., 2011). PER2 is highly expressed in pyramidal cell layers in the hippocampus and its expression fluctuate in circadian manner. Expression of PER2 in the hippocampus is out-of-phase with that in SCN, and PER2 null mice showed abnormal LTP (Figure 3A; Wang et al., 2009). Circadian patterns of expression of CLOCK in the DG and BMAL1 in CA1 and CA3 have been reported in the mouse hippocampus (Wyse and Coogan, 2010). The findings of oscillation of PER1 in the hippocampus are not consistent. The expression of PER1, high in the DG of hippocampus, has not shown to oscillate (Yamamoto et al., 2001; Abe et al., 2002). However, PER1 and BMAL1 in the hippocampus have been shown to oscillate depending on SCN (Wang et al., 2009; Jilg et al., 2010). Interestingly, PER1 has been reported to be upregulated in the mouse hippocampus and cerebral cortex by electroconvulsive shock or kainate injection (Eun et al., 2011). In electrically induced rat model of chronic epilepsy, the excitability of DG shows two distinct phases (high and low) of seizures (Matzen et al., 2012). In the same model, hippocampal CA1 region during latent period of epileptogenesis shows a phase shift between two types of population spikes which follow the circadian rhythm (Talathi et al., 2009).

Suprachiasmatic nucleus (SCN) in the hypothalamus is the central circadian pacemaker to coordinate and synchronize local clocks throughout the body (Welsh et al., 2010). SCN receives direct inputs from tens of different regions, and projects to more than dozen regions which includes three major afferent connections—retinohypothalamic and geniculohypothalamic projections, and median raphe serotonertic pathway (for the detail, see Morin, 2012). A direct neural pathway from the hippocampus to SCN is known, however, the SCN output to hippocampus is still unclear (Krout et al., 2002). It has been reported that the indirect pathways through multiple synaptic connections and hormonal influence (e.g., hypocretin and melatonin) onto the hippocampus confer the circadian rhythmicity (Monnet, 2002; Perreau-Lenz et al., 2003). Subiculum to SCN connection has been reported, and long-range GABAergic projections may be able to synchronize the oscillatory activity between these two areas (Meibach and Siegel, 1977; Canteras and Swanson, 1992; Jinno et al., 2007). As shown in paraventricular nucleus, the strength of the GABAergic input from the SCN to subiculum can be rhythmic (Kalsbeek et al., 2008). Either strong excitatory input or weak inhibitory input from SCN to subiculum/hippocampus, with or without synchronization to the rhythmic excitability of the hippocampus, may overcome the seizure threshold (Figure 3B). Interestingly, hippocampus-dependent spatial and contextual fear memories were compromised when the SCN is lesioned after training (Phan et al., 2011). It will be interesting to see if the circadian episode of seizures will be altered when the SCN of the epileptic animals is lesioned. To study this SCN output pathway to hippocampus transgenic mice with fluorescence labeling of identifiable neuronal population (e.g., GAD-GFP mice) and optogenetic approaches (e.g., Channelrhodopsin2 and Halorhodopsin) may be useful (Adamantidis et al., 2010; Kokaia et al., 2012).

## Involvement of epigenetics in circadian rhythm and epilepsy

Epigenetic regulation should be considered in this type of study because individual organisms show the differential response in both circadian rhythm and epilepsy to environmental stimuli (Bellet and Sassone-Corsi, 2010; Qureshi and Mehler, 2010). It should be noted that the CLOCK protein has a histone acetyltransferease activity (Doi et al., 2006). There are circadian changes in histone acetylation at the promoter of CLOCK genes (Etchegaray et al., 2003). MLL1, a H3K4 methyltransferase, is associates with CLOCK and recruited to promoters of CCGs in a circadian manner, and null mice of SMCX/JARID1c, a H3K4 demethylase, develops epilepsy (Tahiliani et al., 2007; Katada and Sassone-Corsi, 2010; DiTacchio et al., 2011). Even in one type of animal model of epilepsy, epileptic animals may not show the single circadian pattern of epileptic episodes (e.g., diurnal vs. diffuse types). Therefore, there is room to improve or develop better models. Examining the circadian behaviors of existing mutant mice with epileptic seizures to find a suitable model is highly desirable (Yoneda et al., 1993).

## Conclusion

The phenomena of circadian rhythmicity of spontaneous epileptic seizures are evident in human and animal models although there are inconsistency and studies yet to be done in detail. Findings from the literature regarding the circadian regulation and epilepsy were reviewed to formulate the rationale for its molecular mechanism. As one may notice there is no strong evidence to support some premises for the hypothesis proposed here, and there are many more questions than answers on the subject of this article. By testing the hypotheses proposed here; (1) fluctuating activity of activated mTOR signaling molecules and their targets increase the neuronal excitability in the epileptic brain, raising beyond the seizure threshold to display the behavioral seizures. (2) The rhythmic input strength from SCN to hippocampus contributes to synchronizing hyperexcitability which manifests with the epileptic seizures. By addressing this question, hopefully we can have the opportunity to address another mysterious side of epilepsy.

### Conflict of interest statement

The author declares that the research was conducted in the absence of any commercial or financial relationships that could be construed as a potential conflict of interest.

## Abbreviations

4E-BP1: eukaryotic initiation factor 4 binding protein 1
AMPK: AMP protein kinase
CCG: clock controlled gene
CK1/2: Casein Kinase1/2
DBP: albumin D-site-binding protein
EEG: eletroencephalography
EPSP: excitatory postsynaptic potential
GABA: gamma-aminobutyric acid
GFAP: glial fibrillary acidic protein
IRS: insulin receptor substrate
LTP: long term potentiation
mTOR: mammalian target of rapamycin
NSE: neuron specific enolase
PI3K: phosphoinositide 3-kinase
STAT3: signal transducer and activator of transcription-3.
